# Comparison of different hydroxyapatite composites for bone tissue repair: *In vitro* and *in vivo* analyses

**DOI:** 10.22038/IJBMS.2024.78578.16995

**Published:** 2024

**Authors:** Xiaoyu Su, Xiang Si, Yuyang Liu, Nana Xiong, Siyuan Li, Lu Tang, Zheng Shi, Lijia Cheng, Fei Zhang

**Affiliations:** 1 1 School of Basic Medical Sciences, Clinical Medical College and Affiliated Hospital, Chengdu University, Chengdu, 610106, China; # These authors contributed eqully to this work

**Keywords:** Angiogenesis, Chitosan, Hydroxyapatite, Osteoinduction, Sodium alginate

## Abstract

**Objective(s):**

The material used for bone tissue repair needs to be simultaneously osteoconductive, osteoinductive, and osteogenic. To overcome this problem, researchers combine hydroxyapatite (HA) with natural materials to improve properties. This paper compares the effects of angiogenesis and osteogenesis with different composites through *in vivo *experiments and characterization analysis.

**Materials and Methods:**

Chitosan/nHA (CS/nHA) and sodium alginate/nHA (SA/nHA) microspheres were synthesized via reverse-phase emulsification crosslinking and analyzed using scanning electron microscopy (SEM), energy dispersion spectroscopy (EDS), and X-ray diffraction (XRD). Implanted into mouse thigh muscles, their angiogenic and osteogenic potentials were assessed after 8 and 12 weeks through various staining methods and immunohistochemistry.

**Results:**

The mean vascular density (MVD) of CS/nHA, CaP/nHA, and SA/nHA groups was (134.92±35.30) n/mm^2^, (159.09±22.14) n/mm^2^, (160.31±42.23) n/mm^2^ at 12 weeks, respectively. The MVD of the CaP/nHA and SA/nHA groups were significantly higher than that of the CS/nHA group. The collagen volume fractions (CVF) were 34.13%, 51.53%, and 54.96% in the CS/nHA, CaP/nHA, and SA/nHA groups, respectively. In addition, the positive expression area ratios of OPN and CD31 in the CaP/nHA and SA/nHA groups were also significantly higher than those in the CS/nHA group.

**Conclusion:**

The ability of SA/nHA composite microspheres in osteogenesis and angiogenesis is clearly superior to that of the CS/nHA group and is comparable to that of CaP/nHA, which has superior osteogenesis ability, indicating that SA/nHA composite microspheres have greater application prospects in bone tissue engineering.

## Introduction

Extensive bone defects caused by disease or trauma have always been a major problem to be solved in the field of orthopedics, which seriously affect the morphology and function of local tissues and cause great damage to patients. Currently, bone grafting is divided into autologous, allogeneic, and xenografts, which have been the most used strategies for the treatment of bone tissue defects in orthopedic surgery (1). Autologous bone is considered the gold standard for bone grafting because of its excellent bone conduction, bone induction, osteogenesis, available sources, ideal biocompatibility, and three-dimensional structure (2). However, the commonly used bone transplantation has some shortcomings, such as limited source, immune rejection, and implantation failure caused by disease transmission (3, 4). In order to solve the above problems, synthetic bone repair materials have become a research hotspot. They have a wide range of sources and no immunogenicity, which can not only provide structural support for damaged bone tissues, but also integrate with surrounding tissues, which has a certain role in promoting bone repair and regeneration, and provides a new method for repairing a large range of bone defects. The best bone substitute materials for the treatment of bone defects include bone tissue engineering composites represented by hydroxyapatite (HA) (5).

 Since its development, HA has been widely used in bone regeneration due to its high bioactivity and bone conduction and has become an important material for biomaterials and tissue engineering research. Not only stimulation and rejection are absent after implantation in living organisms, but they also have bone conduction, biodegradability, and bone induction properties (6). Therefore, HA is an ideal bone repair material because it has the following three key characteristics: osteoconductivity, osteoinductivity, and osteogenesis. Despite these properties, due to the simple HA material having insufficient toughness, brittle failure, slow degradation, and other problems (7), HA is brittle and has limited application in loading-bearing applications. Thus, HA is often functionalized with other polymers, such as chitosan, to provide the mechanical properties required for an implant in the reconstruction and regeneration of bone tissue for better clinical application. The biocomposite properties were further optimized by the synthesis of nano apatite and porous HA (8). Researchers have found that sodium alginate/nHA (SA/nHA) has better mechanical properties and excellent cell viability than SA alone (9). Chatzipetros* et al*. also confirm that obvious new bone formation could be observed when chitosan/nHA (CS/nHA) was applied to rat skull defects, and angiogenesis, osteoblast proliferation, and collagen fiber proliferation could be under the microscope after staining (10). Furthermore, Soriente *et al*. identified that CS/HA scaffolds can support cell proliferation and differentiation thanks to the important chemical features of CS and the bioactive/osteoinductive properties of HA (11). Also, this scaffold can regulate the production of pro-inflammatory (TGF-β) and anti-inflammatory (IL-4, IL-10) cytokines. After bone defects, it will undergo a healing phase of inflammation, osteogenesis, and bone remodeling: in the first stage, numerous growth factors are released after hematoma formation, and mesenchymal stem cells are stimulated by the growth factor cascade to differentiate into chondrocytes and osteoblasts. The next stage is angiogenesis and braided bone produced by endochondral ossification. The last part is bone remodeling, new lamellar bone will replace the braided bone (12). Based on the fact that bone is highly vascularized tissue, and it relies on the tight connection between blood vessels and bone cells to maintain integrity, so when bone replacement materials are implanted in large bone defects, insufficient angiogenesis often leads to reduced bone formation and poor bone healing (13, 14). Therefore, the key to the successful repair of bone defects is the migration and differentiation of osteoblast precursor cells and vascularization. The interaction between vascularization and osteogenesis is determined by autocrine and paracrine factors (15). Factors such as osteopontin and vascular endothelial growth factor (VEGF), which act bidirectionally on osteoblasts and vascular endothelial cells, can promote new bone formation and angiogenesis (16). Considerable attention has been given to the use of chitosan (CS)-based materials reinforced with inorganic bioactive signals such as HA to treat bone defects and tissue loss (11). Some scholars used sodium alginate (SA) to chelate phosphorus ions and calcium which has a function of promoting osteogenesis (17). In previous studies, the two composites were rarely compared. In order to know which composite material is more conducive to bone regeneration, chitosan/nHA or sodium alginate/nHA, we investigate the differences between the two natural materials that combined with nHA respectively, the optimal nHA natural composite was selected by removing the material implanted in the mouse muscle bag at weeks 8 and 12 and observing its ability to promote vascular and bone formation. Our results showed the ability of SA/nHA composite microspheres in osteogenesis and angiogenesis is obviously superior to that of the CS/nHA group, and is comparable to that of CaP/HA, and it indicated that SA/nHA composite microspheres have wonderful potential in bone repair.

## Materials and Methods

***Preparation of CS/nHA***

To prepare CS/nHA composite microspheres, 25 ml of liquid paraffin with 3% Span (Wiggens WH220-HT, Beijing, China) was heated to 50 °C in a mechanical stirring water bath, serving as the oil phase. Separately, 0.15 g of CS powder and HA powder (M:502.31 g/mol, Ca/P=5:3, short rod-shaped, 20 nm) (Macklin, Shanghai, China) were dissolved in 5 ml of 2% ice acetic acid solution, and ultrasonically mixed (Supmile KQ-500DE, Jiangsu, China) to form the water phase with a 1:1 ratio of nHA to CS. This mixture was then gradually added to the paraffin, stirring continuously for 1 hr to create a stable emulsion. To this, 0.6 ml of 25% glutaraldehyde was introduced for cross-linking, stirring for another hour. The resultant yellow-brown emulsion was then centrifuged at 2000 rpm (Zonkia SC-3616, Anhui, China) for 1 min. The supernatant was discarded, and the sediment was washed thrice with isopropyl alcohol, petroleum ether, and anhydrous ethanol, respectively. Finally, the precipitate was dried at 60 ℃ to yield the CS/nHA microspheres.

***Preparation of SA/nHA***

To prepare SA/nHA composite microspheres, 25 ml of liquid paraffin with 6% Span (Wiggens WH220-HT, Beijing, China) was heated to 50 ℃ in a mechanical stirring water bath to serve as the oil phase. HA powder (Macklin, Shanghai, China) was ultrasonically dispersed in distilled water for 30 min, followed by the addition of 0.15 g sodium alginate (M:216.121 g/mol). This mixture was stirred vigorously for 2 hr to ensure homogeneity. The aqueous phase was then combined with the oil phase and stirred continuously at 60 ℃ for 2 hr in the mechanical stirring water bath, resulting in a stable white emulsion with a ratio of nHA to SA of 1:2. Subsequently, 8% (w/v) CaCl_2_ was gradually introduced to the emulsion for crosslinking and curing over an hour. After adding a small amount of anhydrous ethanol to the emulsion and letting it stand for 10 min, the upper solution was removed. The remainder was centrifuged at 2000 rpm for 1 min (Zonkia SC-3616, Anhui, China), and the supernatant was discarded. The sediment was washed three times with isopropyl alcohol, petroleum ether, and anhydrous ethanol. Finally, the precipitate was dried at 60 ℃ to obtain the SA/nHA composite microspheres.

***Scanning electron microscope (SEM)***

The morphology and particle size distribution of composite microspheres were analyzed using the Regulus 8100 high-resolution field emission SEM. Samples were prepared by drying and gold-sputtering under specific conditions before imaging at 10 kV (18). Microsphere diameters were measured with Image J software.

***X-ray diffraction (XRD)***

The crystal structure and mineral composition of the two composite microsphere samples were further determined. Through XRD, the results were recorded on the XRD (Bruker D8 advance, Karlsruhe, Germany). A Cu target Kα-ray was employed in the analysis (19). The experimental data were compared with the HA standard card (JCPDS standard card PDF-09-0432).

***Energy dispersive spectrometer (*** ***EDS)***

EDS is mostly used in combination with SEM and is widely used to analyze the types of various material components. Using Oxford JSM 6290 EDS to analyze two composite microsphere samples, their element composition can be obtained. Take an appropriate number of samples and fix them on the platform with conductive glue. After vacuum spraying the conductive layer, place them in the sample room. After adjusting to the best observation position, select the sample observation area, take pictures of the microstructure of the sample, and analyze the types and content of elements in the micro area.

***Animal surgery***

Thirty 8-week-old male ICR mice from Dossy Biotechnology (Chengdu, China) were obtained. All animals were maintained in a temperature- and light-controlled environment ventilated with filtered air. Animals were randomly divided into three groups (n=10). Intraoperatively, mice were anesthetized with 3% pentobarbital sodium injection (1 mg/100 g body weight). SA/nHA, CS/nHA, and CaP/nHA (Volume 1 cubic centimeter) were sterilized prior to implantation. Thigh hair was removed using an electric razor, and the skin was sanitized with ethanol before making an incision. Using ophthalmic scissors, a precise 2 cm long incision was made through the skin and deep fascia at the midpoint of both thighs (20). The muscles surrounding the posterior part of the femur were delicately separated to reveal the lateral aspect of the thigh’s femur. This careful dissection facilitated the creation of a pocket between the skin and the lateral posterior part of the femur, intended for the implantation process. SA/nHA, CS/nHA, and CaP/nHA composite microspheres were filled into muscle pockets in the muscle pockets of different mice, and the muscle pockets of both thighs of one mouse were filled with the same material. The materials were uniformly converted into the same volume and implanted into the mice. The CaP/nHA group was set as the positive control group. Finally, the incision muscle and skin were sutured with nylon sutures, and penicillin, 40 mg, was intramuscularly injected to prevent infection. All animals were then cultured in a sterile chamber at constant temperature. The cervical vertebrae of the animals were sacrificed at different time points in the 8^th^ and 12^th^ week, and the activity of bone-inducing substances was evaluated. All animal experimental protocols were approved by the Animal Ethics Committee of Chengdu University. The most important procedure and animal care is to comply with the National Institutes of Health guidelines on the care and use of laboratory animals under the supervision of a licensed veterinarian.

***Histological staining***

At 8 and 12 weeks postoperatively, animals were killed and specimens were collected. The samples were immediately fixed in 4% paraformaldehyde at room temperature for 48 hr, then replaced with Ethylene Diamine Tetraacetic Acid (EDTA) decalcified solution in a constant temperature water bath shaking bed, and replaced with fresh decalcifying agent every three days until there was no resistance to the puncture needle going into the tissue, and finally embedded in paraffin wax. The embedded specimen was cut into histological sections 5 μm thick. The sections were stained with Hematoxylin-Eosin staining (HE), Masson, Safranin O-Fast green staining, and Sirius Red staining.

***Immunohistochemical staining***

The Platelet Endothelial Cell Adhesion Molecule-1 (CD-31) (Shanghai Jianglai Biotechnology Co., LTD, China) and Osteopontin (OPN) (Shanghai Jianglai Biotechnology Co., LTD, China) were selected for immunohistochemical staining. CD-31, as a specific marker of vascular endothelial cells, is commonly used to evaluate neovascularization. The cytoplasm of CD-31 positive cells is light yellow or brownish yellow and microvascular. OPN is a glycosylated protein, which is mainly expressed in the late stage of bone formation and widely exists in the extracellular matrix. It is also an important bone matrix protein, which is closely related to the formation and development of bone. The number of neovascularization and bone cells in the section was observed.

***Morphological analysis***

Image J software was used to calculate the collagen area ratio of the samples at weeks 8 and 12 as collagen area/total tissue area, and the average blood vessel density at week 12 was calculated as the number of blood vessels/corresponding tissue area under 200 times the visual field. During the analysis, the three slices of the scan were analyzed respectively, and the values were the average of the results of the three slices.

***Statistical analysis***

Abnormal value could be observed according to the principle of 3σ. Since all of the statistics were calculated by 2 different people through the Image J software, and have practical significance for the results, the abnormal value was retained for statistics analysis. Each group has five samples. One-way analysis of variance (SPSS 22.0, USA) was used to present the data in the form of mean ± standard deviation. *P*<0.05 was considered a significant difference.

## Results

***Microscopic morphology of the materials***

**Figure 1:**
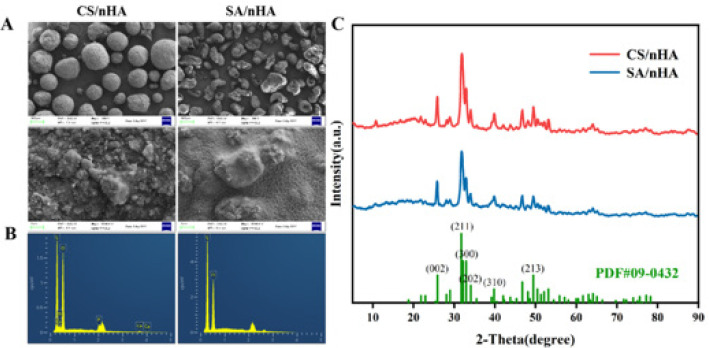
Results of material characterization

The morphology and dispersion of the completed CS/nHA composite beads are shown in Figure 1, and the average diameter of CS microspheres was 156.26±33.83 µm, and that of alginate microspheres was (97.53±29.18) µm. The sphericity of the A composite microspheres tends to be regular, the dispersion of nHA on the surface is relatively uniform, and the adhesion between particles is small. The particle size distribution of SA/nHA composite microspheres was wide, and the dispersion of nHA on the surface of SA/nHA microspheres was less uniform than that of CS/nHA. The dispersion is poor, the surface roughness of the microspheres increases, and the short rod-shaped nHA particles can be seen after magnification, and they begin to appear “wrinkled”. This is due to the small specific gravity of nHA in the microspheres which is uniformly coated inside the microspheres, which has little effect on the morphology of the microspheres, but when the content of nHA is more than SA, the characteristics of nHA itself are brittle and have poor strength, so that the surface of the microsphere becomes loose and the pellet formation is poor. EDS can only measure the content of the surface, and the surface of the SA/nHA microspheres is uneven, irregular in shape, and scattered with agglomerated uneven nHA, which may be the reason why the Ca peak of SA/nHA in the EDS spectrum is not measured. Moreover, SA could absorb the X-rays of the light element calcium in the vicinity, which has a low yield, may result in less Ca content in the selected area, and the Ca peak is not visible. 

***Material composition***

According to JCPDS standard card PDF-09-0432, XRD standard spectral analysis was used to further study the composition and crystallinity of CS/nHA composite microspheres. As can be seen from Figure 1 C, the XRD spectra of the two composite microspheres at 2θ=25.9°, 31.9°, 32.2°, 33.0°, 34.1°, and 39.9° have the same diffraction peak intensity as nHA, and no other impurity peaks appear. CS has characteristic peaks at 20.2° (21), and SA has a strong peak at 31.7° (22), neither was represented in the XRD patterns, which could be explained by the low crystallinity that is derived by interactions between HA, SA, and CS, a state that is similar to natural bone. Moreover, all the recorded spectra were close to HA, which may be due to the higher amount of HA, so the main peak contributed to HA. 

***Histological staining***

**Figure 2:**
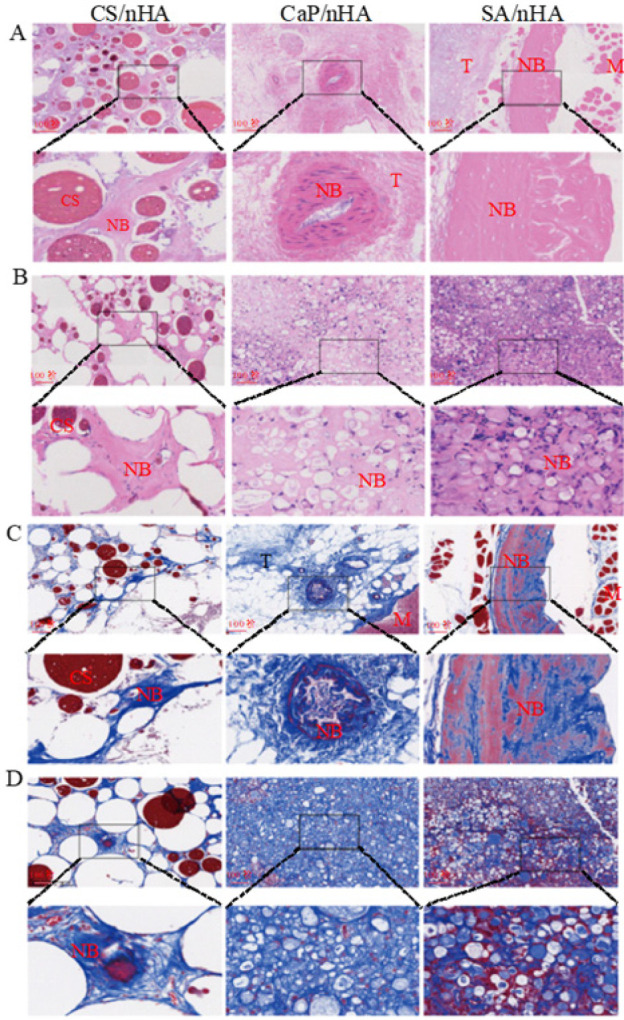
HE and Masson-stained light micrographs of 8-week-old male

**Figure 3:**
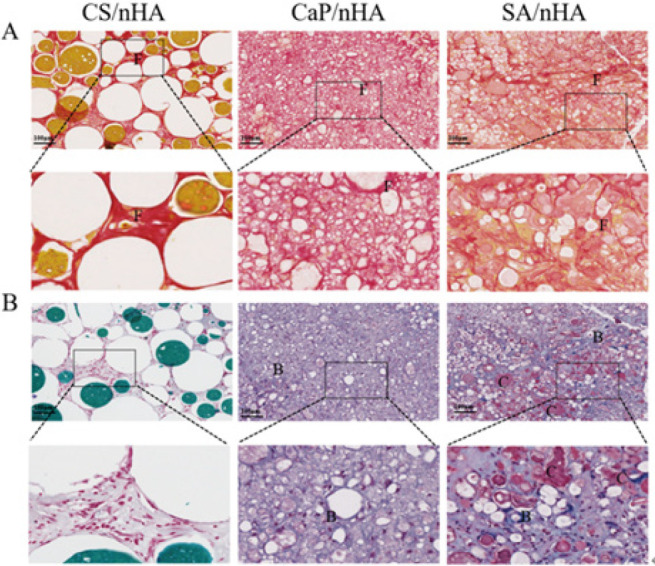
Microscopic images of Sirius red staining and Safranin and fast green staining staining in 8-week-old male ICR mice implanted with CS/nHA, CaP/nHA, and SA/nHA materials at 12 weeks

The samples implanted with materials for 8 weeks were examined using HE and Masson staining (Figure 2). In the SA/nHA group, significant bone regeneration was observed, along with evident bone lacunae structures in the newly formed bone. Masson trichrome staining revealed a large area of red-stained new bone, indicating relatively mature bone tissue generation at 8 weeks in the SA/nHA group. Conversely, the CaP/nHA group exhibited only bone-like tissue, while the CS/nHA group showed limited new bone formation and a substantial amount of undigested chitosan material, which can be seen in Figure 2C. At 12 weeks, both SA/nHA and CaP/nHA groups displayed extensive areas of new bone compared to the CS/nHA group in Figure 2B, D, and Figure 3. Through image J calculation, it also was determined that the collagen area ratios of the SA/nHA and CaP/nHA groups at 12 weeks were significantly higher compared to those of the CS/nHA group. At 12 weeks, the collagen area ratio of the SA/nHA group (51.53±6.45) % was comparable to that of the CaP/nHA group (54.96±1.96) % but substantially higher than that of the CS/nHA group (34.13±8.87) % (Figure 5A ). The Sirius red staining area ratio in the SA/nHA group (39.29±6.31) % was similar to that in the CaP/nHA group (40.08±5.59) %, yet considerably greater than that in the CS/nHA group (14.42±2.63) % (Figure 5B). These findings suggest that the SA/nHA group exhibits stronger early osteogenic ability but similar final osteogenic capacity as the CaP/nHA group. Sirius red staining demonstrated orange-red collagen fibers under an optical microscope, with large regions of red collagen fibers observed in both SA/nHA and CaP/nHA groups; however, their specific type could not be determined. Combined saffron green staining revealed mature green-colored bone tissue and red-colored cartilage tissue in the SA/nHA group.

***Immunohistochemical staining***

**Figure 4:**
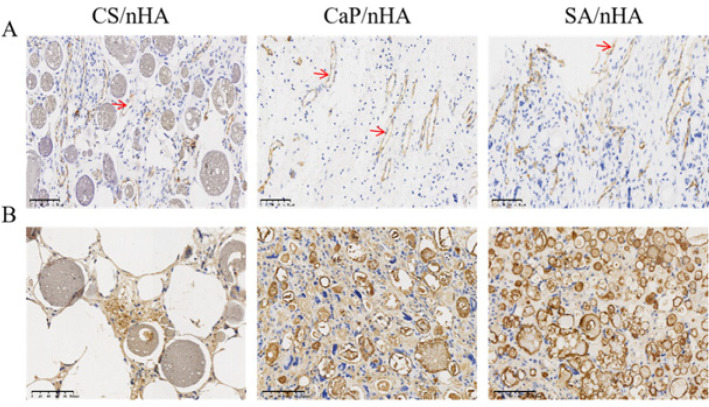
The specific expression of CD-31 and OPN in 8-week-old male ICR mice after implantation of CS/nHA, CaP/nHA and SA/nHA materials

**Figure 5:**
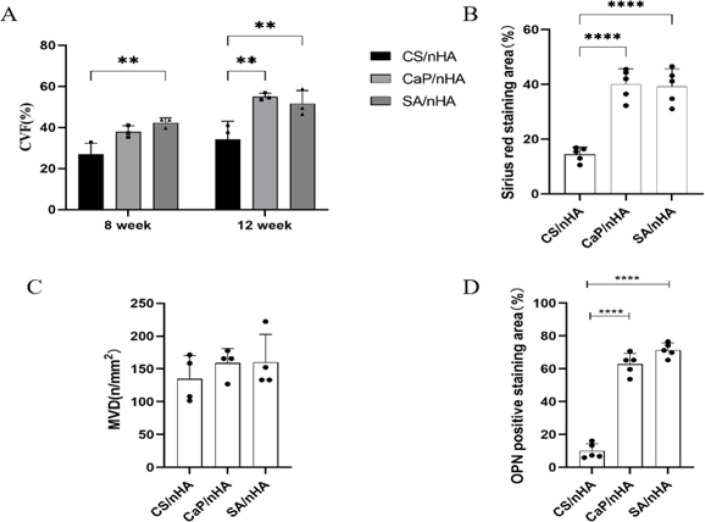
(A) Statistical plot of collagen area ratio of 8-week-old male ICR mice at 8 and 12 weeks after surgery; (B) Sirius red collagen fiber area ratio at week 12; (C) Mean blood vessel density at 12 weeks; (D) OPN positive area ratio data at 12 weeks

The specific expression of CD-31 on vascular endothelial cells was observed in all three groups. As can be seen in Figure 4A, the expression of brown-stained strip vessels by vascular endothelial cells was not significantly different between SA/nHA, CaP/nHA, and CS/nHA groups. However, The quantitative data confirmed that while there was no significant difference between average vascular densities of SA/nHA (160.31±42.23) n/mm^2^ and CaP/nHA (159.09±22.14) n/mm^2^ groups, they were notably greater compared to that observed for the CS/nHA group (134.92±35.30) n/mm^2 ^(Figure 5C), which confirmed superior bone regeneration ability for the SA/nHA group as compared with both CaP/nHA and CS/nHA groups, with blood vessel count potentially playing a crucial role.

The OPN-specific expression of the brown area in the SA/nHA and CaP/nHA groups was significantly higher than that in the CS/nHA group. The OPN positive area ratio in the SA/nHA group (12.80±2.38) % was slightly higher than that in the CaP/nHA group (10.180±0.63) %, and significantly higher than that in the CS/nHA group (1.89±1.19) % (Figure 4B, 5D).

## Discussion

Using SEM to characterize the composite microspheres of CS/nHA and SA/nHA, it can be observed that the composite microspheres combined with CS and nHA have a more orderly shape and more uniform particle size, and the HA on the surface of SA composite microspheres is rod-like and rougher. The crystal structure and elemental composition of the material were verified by XRD and EDS, respectively. Neither the SA special diffraction peak of XRD nor the SA content in EDS detection was obvious, because of the higher amount of nHA, so the main peak in the spectrum is attributed to nHA (23). 

*In vivo *ectopic osteogenesis experiments can directly and comprehensively evaluate the osteogenic activity of the material. Li *et al.* studied the osteogenic activity of demineralized bone matrix (DBM) by implanting the material into the mouse muscle pouch to evaluate the osteogenic effect of DBM (24). As a natural polymer, its unique three-dimensional gel structure mainly provides a good environment for cell proliferation (25). Zhang* et al*. proved in vitro that SA could support the process of cell adhesion and proliferating (25), and Pravdyuk *et al*. even placed SA in a freezing environment to maintain the activity of MSCs (26). In bone tissue engineering, SA and CS are often combined with other materials in order to improve the osteogenic ability (26, 27). Researchers demonstrated good osteogenic performance of Calcium Phosphate Cement-SA scaffolds (28). Because of its good biological properties, SA is mostly used to simulate the organic phase of bone and the composite application of nHA, which is similar to the inorganic phase of bone (29). Luo (30), Chae (31,32), and Yu (32) *et al.* all confirmed that HA-SA composite scaffolds have good cytocompatibility and adhesion for osteogenesis, and another study (33) also verified that the combination of HA and CS can also improve the osteogenic performance by enhancing bone conduction. Soriente *et al.* concluded through experiments that CS-HA not only has good osteogenic ability, but when HA content is greater than 50%, HA content has a greater impact on osteogenic ability (11). A study implanted CS-SA scaffolds into mouse muscle pockets. Twelve weeks later, there was still no obvious bone tissue formation, but a large number of collagen fibers were scattered around and associated with angiogenesis (34). The data of Vasilyev *et al.* also showed that bone conduction ability was related to angiogenesis (35). The staining results of HE and Masson showed that the bone formation ability of SA was significantly greater than that of the CS group, the HA content of the two groups was the same, and the CVD of the SA group was significantly greater than that of the CS group, indicating that the angiogenesis ability affected the bone formation ability of materials. In addition, observation of sections showed that there was always a large amount of undegraded CS in the sections of the CS group. Gel dissolution is crucial for new bone formation, as it can provide growth space for cells to take the opportunity to organize and form new bone (36). At first, researchers implanted chitosan into a defect in the femoral condyle of sheep for 20 days, and a large amount of CS remained undegraded, which was thought to be due to the low degradation rate of CS (37). The higher the degree of deacetylation, the lower the degradation rate (38). The degree of deacetylation of chitosan used in this study was lower. Therefore, the weak ability of CS to compose bone may be related to the angiogenic ability and degradation ability.

(A) Photos of micromorphology of CS/nHA and SA/nHA composites at two different magnifications. Bar above: 100 µm; bar below: 1 µm. (B) EDS energy profiles of CS/nHA and SA/nHA composite microspheres. (C) Surface XRD spectra of the CS/nHA and SA/nHA are compared with standard HA

CS/nHA: Chitosan/nHA; SA/nHA: sodium alginate/nHA; EDS: energy dispersion spectroscopy;XRD: X-ray diffraction; HA: hydroxyapatite

ICR mice after implantation of CS/nHA, CaP/nHA, and SA/nHA materials 8 and 12 weeks postoperatively

HE staining at 8 weeks (A) and 12 weeks (B) showed that obvious mature bone tissue could be observed in the SA/nHA group, osteoid formation was observed in the CaP/nHA group, and large areas of mature bone tissue were scattered in both groups at 12 weeks; Masson staining at 8 weeks (C) and 12 weeks (D) showed that large areas of collagen fiber precipitation were observed in SA/nHA and CaP/nHA groups, and mature bone tissue could be observed in the SA/nHA group at 8 weeks.

NB: New bone; T: Tissue; M: Muscle. bar: 100 μm; CS/nHA: Chitosan/nHA; SA/nHA: sodium alginate/nHA

(A) Sirius red staining showed large areas of red collagen fibers in the SA/nHA and CaP/nHA groups. (B) Safranin and fast green staining showed that in the SA/nHA group, in addition to green mature bone tissue, a large area of red cartilage tissue.

F: Collagen fiber; C: Cartilage; B: Bone. bar:100 μm; CS/nHA: Chitosan/nHA; SA/nHA: sodium alginate/nHA.

12 weeks after surgery

(A) Specific expression of CD-31 (arrow: blood vessel); (B) Specific expression results of OPN (brown: positive expression). bar:100 μm.

OPN: Osteopontin

OPN: Osteopontin

Statistics were expressed as mean ± standard deviation, n=5, **P<0.01; ****P<0.001

## Conclusion

Combining the above content, we can conclude that SA/nHA composite microspheres have better performance than CS/nHA. As a bone repair material, the SA/nHA composite microspheres prepared in this study not only have good microsphere morphology, uniform crystal size, and less agglomeration but also can be compared with CaP/nHA in promoting angiogenesis and osteogenic ability. It provides a theoretical and practical basis for further exploring the application of SA/nHA composite microspheres in bone tissue defect and bone regeneration, *etc.*, and has broad application prospects in the field of biomedical application. With the continuous development of advanced materials and further clarification of the metabolic mechanism of the bone microenvironment, the next generation of bone repair materials will not only have basic bone conductivity, bone induction, and vascular induction but also integrate and regulate different biological activities, so that the degradation of materials* in vivo* and bone repair can reach a perfect match, and reconstruct and even accelerate the process of bone repair under physiological conditions.
